# Cost of dialysis in Tanzania: evidence from the provider’s perspective

**DOI:** 10.1186/s13561-015-0064-4

**Published:** 2015-10-13

**Authors:** Lawrencia Mushi, Markus Krohn, Steffen Flessa

**Affiliations:** Department of Health Care Management, University of Greifswald, Faculty of Law and Economics, Friedrich-Loeffler-Str. 70, D-17489 Greifswald, Germany

**Keywords:** Cost analysis, Hemodialysis, End-stage renal disease, Tanzania

## Abstract

**Background:**

Although End Stage Renal Disease (ESRD) is a disease of increasing epidemiological relevance very little is known about the cost of providing the respective dialysis services in Tanzania. This paper estimates the costs of dialysis for ESRD patients at Muhimbili National Hospital (MNH) in Tanzania in the year 2014.

**Methods:**

Cost calculations are based on the provider perspective and include only the direct cost of dialysis treatment. Cost of drugs and consumables were obtained from the price list issued by the Medical Stores Department (MSD) in Tanzania. Additional data were collected through face-to-face interview with experts at the dialysis unit.

**Results:**

MNH performs on average 442 hemodialysis per month (34 patients, with three sessions per week) with a personnel placement of 20 nurses, four nephrologists, eight registrars, one nutritionist, two biomedical engineers, four health attendants and nine dialysis machines. The respective average unit cost per hemodialysis is 176 US$. Consequently, an average patient requiring three dialyses per week (i.e. 156 dialyses per year) will cause annual costs of 27,440 US$.

**Conclusion:**

The cost of dialysis is enormous for a least developed country like Tanzania where resources and technology are rather limited. Thus, from the economic point of view, it seems rational to allocate health care budgets towards diseases that are curable, have a higher cost-effectiveness and cater for the majority of the population. However, before a final decision on allocation of budgets towards dialysis is made all effort must be invested to improve technical efficiency by cutting the enormous unit cost.

## Background

The incidence and prevalence of chronic kidney disease (CKD) has significantly increased in recent years in both developed and developing countries. This disease is consuming a huge proportion of health care finances in developed countries, while contributing significantly to morbidity, mortality and decreased expectancy of life in the developing world [1, 2]. CKD is increasingly recognized as a global public health problem and as a key determinant of poor health outcomes [3]. However, CKD prevention programs are rare in the developing world. Contrary to some other chronic diseases such as cardiovascular diseases, diabetes, chronic respiratory disorders, and cancer, CKD has not received the same degree of attention [1].

End stage renal disease is an irreversible loss of glomerular filtration rate resulting from a chronic worsening of renal function from chronic renal diseases to end-stage renal failure. Incidence and prevalence of End Stage Renal Disease (ESRD) requiring renal replacement therapy (RRT) are increasing worldwide. It is estimated that by the year 2030, more than 70 % of the patients with ESRD will be residents of developing countries, whose collective economies will account for less than 15 % of the total world economy [1]. The high burden of ESRD and associated costs, related adverse outcomes, and decreased productivity make it a significant public-health problem worldwide [4].

In absence of renal transplantation (as it is the case in many least developed countries) patients with ESRD depend on RRT by renal dialysis treatment for the rest of their lives. The availability and quality of a dialysis program largely depend on the prevailing economic conditions, the political social structure, overall health care facilities and the health care financing strategies. For the ESRD patient in economically advanced countries, the focus now is on improving quality of life and increasing long-term survival. In marked contrast, developing countries are grappling with the short-term patient survival and the enormous cost of therapy that limit continuation of treatment in the majority of patient with ESRD [5].

The prevalence of ESRD in Sub Saharan Africa is reported to be less than 100 per million [1]. However, the credibility of statistics from many developing countries is questionable, and the majority of experts suggest that 150 per million populations is a more realistic estimate of the incidence of ESRD [6]. Irrespective of the tremendous epidemiological relevance very little is known about the cost of dialysis for hemodialysis (HD) in Sub Saharan Africa and estimates have a wide range. In a recent review (Mushi L, Marschall P, Flessa S, The cost of dialyis in low- and middle-income come countries. Submitted.) found only ten studies on the cost of hemodialysis in Sub-Saharan Africa. They were from six countries and had extremely diverse figures Table 1 shows the results.

**Table 1:** Cost of Dialysis in Sub-Saharan Africa

Country	Author(s) and year	Annually cost per patient [Int$ 2012]
Sudan	Abu-Aisha and Elamin 2010 [8]	11,060
Sudan	Elsharif, Elsharif et al. 2010 [9]	15,280
Senegal	Abu-Aisha and Elamin 2010 [8]	28,430
Kenya	Abu-Aisha and Elamin 2010 [8]	16,850
Namibia	Abu-Aisha and Elamin 2010 [8]	25,800
Nigeria	Okafor and Kankam 2012 [10]	42,800
Nigeria	El Matri, Elhassan et al. 2008 [11]	19,700
Nigeria	Abu-Aisha and Elamin 2010 [8]	36,320
South Africa	Abu-Aisha and Elamin 2010 [8]	7370
South Africa	El Matri, Elhassan et al. 2008 [11]	24,880

Source: Mushi L, Marschall P, Flessa S, The cost of dialyis in low- and middle-income come countries. Submitted.

For instance, the annual cost per HD patient are calculated as US$ 7000 in South Africa [7] and range between US$ 25,000-55,000 in Nigeria [7]. Peritoneal dialysis (PD) costs range between US$ 7000 in Egypt [8] and between US$ 20,000-49,000 in Nigeria [7].

Etiology of ESRD in the sub region in Sub-Saharan Africa differs from that seen in the developed world. As opposed to the situation in Europe and the United States, where diabetic nephropathy constitutes close to 50 % of patients on ESRD programs, the predominant causes of ESRD in Africa are essential hypertension and chronic glomerulonephritis [9]. Literature reports the management of ESRD in low and middle-income countries to be too expensive, and health care resources and budgets are unable to meet the burden of treatment [10]. However, lack of studies in least developed countries makes it difficult to understand the cost of dialysis in these countries so that any allocation of health care resources cannot be evidence-based.

To our knowledge, dialysis services were never costed in Tanzania. This paper intends to bridge this gap by reporting the cost of dialysis in Tanzania derived from Muhimbili National Hospital (MNH), the highest public referral hospital. With this data we want to support planners and policy makers to make an evidenced based decision concerning the allocation of scarce resources and the provision of dialysis treatment in Tanzania. Consequently, this paper provides the first estimate of unit and annual cost of dialysis in Tanzania.

## Methods

### Research object

Tanzania is a least developed country [11] with a gross national product per capita (p.c.) of 695 US$ (2012) and health expenditure of 41 US$ p.c. (2012) [12]. For the fiscal year 2012–2013 it allocated 10 % of its national budget on health care services, which is below the standard of the Abuja declaration of 15 % [13]. 40 % of health care expenditure is public with a mix of resources from National government, Regions, Districts, and Development Partners. A smaller group of patients are covered by the National Health Insurance Fund (NHIF) or private health insurances [14] but the majority of patients have to bear a heavy load of out-of-pocket (OOP) expenditure [15].

The prevalence and incidence of CKD in Tanzania are not known as there are no national registries for this disease. However, an alarming high prevalence of CKD (87.3 %) among Tanzanian adult attending diabetes mellitus clinic of Bugando medical centre in Mwanza Tanzania has been identified [16]. Renal transplantation is not performed in Tanzania and only few patients can afford going abroad for an operation. Thus, hemodialysis is the main form of Renal Replacement Therapy (RRT) available in Tanzania. Currently, it is offered at three public and nine private hospitals. 75 % of these service providers and a considerable – but unknown – share of dialysis machines are concentrated in Dar es Salaam, the former capital of the country. Thus, the majority of Tanzanians have no access to dialysis services due to the high concentration in one city.

Furthermore, the majority of patients in Dar es Salaam face severe financial barriers to access dialysis services. As only a minority of the population is covered by NHIF or private insurances, the majority of ESRD patients in Tanzania would have to pay OOP for dialysis services. In a least developed country like Tanzania where 28.2 % of the population is below the official poverty line [12] and the vast majority of patients is vulnerable to poverty as soon as they fall chronically ill dialysis patients are always at risk of losing their lives because they cannot afford dialysis on the free market.

The study was conducted from January to March 2014 at the hemodialysis unit of the MNH in Dar es Salaam. With a population of 4.36 million this city accounts for some 10 % of the total Tanzania Mainland population [17]. MNH is a national referral and university teaching hospital with 1500 beds and some 1000 to 1200 outpatients per week. The dialysis unit at MNH is owned and run by the government. It performs HD sessions with 10 dialysis beds in 3 shifts per day and 6 days a week. Thus, the dialysis unit has a theoretical average capacity of 780 dialyses per month. The standard treatment requires 4 h per dialysis and should be performed thrice per week and patient. The unit provides dialysis services for NHIF members and for acute patients who are exempted.

### Data sample and cost categories

We took a provider perspective and calculated the cost of dialysis based on provider expenditures. During the study period there were 34 hemodialysis patients who underwent outpatient dialysis treatment. All of these patients with 442 dialyses per month were included in the analysis.

The objective of this study is not only to determine the actual average cost per dialysis but also to produce a cost function that allows the analysis of the impact of changing parameters. For that purpose we could not simply divide the total cost during the study period by the number of dialysis but had to develop a cost function that distinguishes between variable, fixed and step-fixed. For the costing of services we follow a standard methodology which has been frequently applied in the calculation of unit costs of medical procedures (e.g. [18]) and which has also been applied to the costing of dialysis services in developed [19] and developing countries [20].

As variable cost we defined all materials and medications that can be classified directly to one hemodialysis. These costs increase proportionally with the number of dialysis [21]. The main variable costs are the dialyzer, bicarbonate concentrate, blood line, normal saline, syringes, gauze sterile, needles, adhesive wound plaster, spirit, iodine, sterile gloves, disposable mask, apron, bed sheets and the anticoagulation heparin. Although Erythropoietin is sometimes given during the procedure, it was excluded from the costing as it is a consequence of the disease and not directly caused by the dialysis.

The cost for each medication, material and the water was determined to the best possible degree of precision. The cost of dialysis water was obtained by calculating water consumption of a single dialysis procedure multiplied by price per liter according to Dar es Salaam Water and Sewerage Corporation rates, including sewerage charges and value added tax. Prices for the materials and medication were collected from the price list issued by the MSD in Tanzania.

Other costs that clearly increase with the number of dialysis are costs for electricity, waste management, laboratory and sterilization. The cost of electricity per month was calculated with the help of the electrical engineer of the hospital. The total energy consumption per month was calculated taking into account all electrical appliances at the unit, watts consumed by each appliance, duration of usage of appliances per day and number of days the unit operates per month. The monthly electricity consumption thus calculated was then used to calculate the monthly electricity cost according to standard Tanzania Electric Supply Company (TANESCO) rates. Sterilization cost was calculated with the help of the head of the respective department, identifying and adding together the number of sterilized packs multiplied by the unit price to obtain total sterilization cost per month. In addition, waste management costs were also calculated and obtained by counting the total number of waste disposal buckets and the total number of plastic bags used per day multiplied by the unit price to obtain the total waste cost spent per month. Laboratory cost was calculated by adding the costs for all tests carried out during a month. Referring to the average of 442 dialyses per month the variable cost per dialysis was defined for the four categories, so that it is possible to add them directly to a single dialysis.

The costs for building rent, water quality management, service for water treatment plant and administration are indirect, as they cannot be allocated directly to one service unit. Thus, they do not vary with the number of dialysis and are fixed costs. Building rent of the dialysis unit was calculated with the help of the estate manager. We measured the dialysis unit surface per square meter. The total square meter was then multiplied by the unit price per square meter according to Tanzania Revenue Authority property tax rates to obtain the monthly rent cost of the renal unit.

Step-fixed costs are defined as costs that are fixed within a range; otherwise – if the volume exceeds the range – the costs increase stepwise [22]. The cost category includes costs for personnel, dialysis machines depreciation as well as maintenance and repair of the machines. The personnel of the MNH consist of medical staff (physicians, nurses, and nutritionist) and non-medical staff (biomedical engineers and health attendants). The personnel costs were based on gross salaries and obtained from the chief accountant of the hospital. To calculate costs per dialysis depending on the number of dialysis per month it was necessary to determine capacities for each staff category and for the dialysis machines. Based on the average number of dialysis per month, the staff level, the regular working hours, the percentage of working time on the dialysis unit, the need of machine maintenance and repair and on the hypothesis that the staff is working on their maximum capacity level the following monthly capacities were obtained (Table 2). In case of an employment full-time equivalent less than 1.00 the monthly cost for the staff category were multiplied with the appropriate full-time equivalent to include only dialysis related costs. Depreciation per machine was calculated with a life time of eight years.

**Table 2:** Monthly treatment capacities per staff member or machine

Cost Category	Capacity [dialyses per month]	Employment full-time equivalent [share]
Dialysis machine	52	-
Nurse	23	1.00
Nephrologist	111	0.70
Registrar	56	0.31
Nutritionist	442	0.50
Biomedical Engineer	221	1.00
Health attendants	111	1.00

Source: Muhimbili National Hospital (MNH)

To calculate the average actual full cost per dialysis depending on the monthly number of dialyses the following formula was applied:

$$ {C}_t=\frac{C_f+{\displaystyle {\sum}_{i\in K}{d}_i*\kern0.5em \left(1+ trunc\left(\frac{x_t}{k_i}\right)\right)}+{x}_t*\kern0.5em v}{x_t} $$

with

- *C*_*t*_ average cost per dialysis in US$
- *C*_*f*_ fixed costs per month in US$
- *d*_*i*_ monthly costs of unit i in US$
- *x*_*t*_ number of dialysis done in period t
- *k*_*i*_ monthly capacity of unit i in US$
- *v* variable costs of one dialysis in US$
- *K* set of step-fixed cost units
- *p* Fee per dialysis in US$

All monetary estimates were provided in Tanzania Shilling (TZS) and converted to US dollar with official 2013 exchange rate published by The World Bank of 1600.44 (1 US$ = 1600.44 TZS) [23]. The cost of materials and medications were derived from the unit prices. The data were obtained through personal measurements as well through face-to-face interviews with experts at the dialysis unit, with key stakeholders of NHIF headquarters and officials of the Ministry of Health and Social Welfare (MoHSW) in Tanzania.

In section 3 we will also present a break-even analysis. This standard methodology of business administration [24] calculates the output quantity that is necessary to recover the full costs. As a production process usually entails fixed costs a low output level will result in a deficit. If the variable cost is lower than the respective revenue per production unit the deficit will decrease with growing production. The break-even point is exactly the output level where production leaves the deficit and starts generating a profit. A rational provider must, thus, safeguard that he can produce and sell at least this number of output units.

This analysis follows the standard methodology of break-even-analysis. However, as the cost function includes step-fixed costs the surplus/deficit curve is not straight but a zig-zag curve, i.e., the break-even point is the solution for the equation:

$$ p\cdot {x}_t-\frac{Cf+{\displaystyle {\sum}_{i\in K}{d}_i}*\kern0.5em \left(1+ trunc\left(\frac{x_t}{k_i}\right)\right)+{x}_t*\kern0.5em v}{x_t}=0 $$

It has to be stated that the objective of this analysis was the calculation of the actual full costs. Thus, we recorded the reality of dialysis services in this particular hospital and calculated the costs of real-life. Consequently, standard costs based on international standards of dialysis services might be higher (e.g. if the standards require more resources than applied in Tanzania) or lower (e.g. if the standards require less resources than applied in Tanzania).

## Results

### Patients’ characteristics

Table 3 describes patient’s characteristics of 34 patients included in our study. Of the study patients, 68 % were male. The majority (74 %) was in the age-set of 15–49 years, about a quarter (23 %) was in the age-set 50–69 years and only a few patients (3 %) were in the oldest age set (70–89). Causes of ESRD were mainly due to chronic glomerulonephritis, which accounted for 64 % of the study population. Diabetes nephropathy accounted for (13 %), hypertension for 10 %, polycystic kidney disease for 10 %, and atypical hemolytic uremic syndrome rinse for 3 %. For Acute Kidney Injury (AKI) causes were rapidly progressive glomerulonephritis (67 %) and placenta abruption (33 %). In addition, patients had different types of vascular access including catheter, arteriovenous (AV) fistula and AV graft. Among them, 62 % had a catheter as an access point, 35 % had AV fistula and the rest 3 % had AV graft.

**Table 3:** Characteristics of the study patients

Variable		Total Number of patients (34)	Percentage
Gender			
	Male	23	68
	Female	11	32
Age distribution			
	15-29	8	24
	30-49	17	50
	50-69	8	23
	70-89	1	3
Causes of ESRD		31	
	Chronic Glomerulonephritis	20	64
	Diabetes nephropathy	4	13
	Hypertension	3	10
	Polycystic	3	10
	Atypical hemolytic uremic syndrome rinse	1	3
Causes of AKI		3	
	Rapidly progressive glomerulonephritis	2	67
	Placenta abruption	1	33
Type of vascular access			
	Catheter (jugular, subclavian, femoral)	21	62
	Arteriovenous fistula	12	35
	Arteriovenous graft	1	3

Source: Muhimbili National Hospital (MNH)

### Cost analysis

Tables 4, 5 and 6 show the cost categories for fixed, variable and jump-fixed costs. The fixed cost of the dialysis department is 2990 US$ per month and the variable cost per dialysis is 80 US$. The strongest cost driver is the dialyzer high flux with 25 US$ and the sterilization with 13 US$. Table 2 exhibits the capacities of the resources with jumped-fixed costs, the respective unit costs are expressed in Table 6. It has to be noted that the costs of personnel do not reflect their net salary but the total cost for the employer, including all allowances, pension fund, insurance, garments etc. In particular the cost per nurse seems to be high but these are specialized nurses with academic degrees who are scarce in Tanzania.

**Table 4:** Fixed Cost

Cost category	cost per month [US$]
Rent/month	2455.57
water Quality mgt	418.63
servicing of water treatment plant	53.01
Administration	62.98
sum of fixed cost per month	2990.20

source: Muhimbili National Hospital (MNH)

**Table 5:** Variable Cost

Cost category	cost per dialysis [US$]
Dialyzer high flux	24.51
Bibag 1	4.37
Blood line	6.25
Nornal saline 500mls (2x)	0.31
Syringes 20 cm^3^	0.09
Syringes 10 cm^3^ (4x)	0.27
Syringes 5 cm^3^ (4x)	0.15
Iv giving set	0.94
Heparin 5000 I.u	4.37
Gauze sterile 1roll 40000	3.12
Adhesive wound plaster 1pair	0.69
spirit 20mls	8.12
Iodine providine 20mls	1.56
Sterile gloves (pair) (4x)	0.40
clean gloves (pair) (4x)	0.40
Disposable mask (2x)	0.62
safety boxes	0.62
Plastic Apron disposable	1.25
water cost (120 l per dialysis)	0.11
Electricity cost	3.29
waste	0.06
laboratory	0.57
laundry (4sheets per dialysis)	5.00
sterilization	13.12
sum of variable (per dialysis)	80.21

Source: Muhimbili National Hospital (MNH)

**Table 6:** Jump-Fixed Cost

Cost category	unit cost per month [US$]
depreciation dialysis machine	159.46
maintenance	24.99
dialysis nurse	1178.74
nephrologist	1951.34
registrar	1602.68
nutritionist	1471.16
biomedical engineer	1341.51
health attendant	317.26

source: Muhimbili National Hospital (MNH)

Based on the number of 34 patients with 442 dialysis per month the average cost per dialysis is 176 US$. This includes the apportioned costs for 20 nurses, four nephrologists (full-time equivalent 2.80), eight registrars (full-time equivalent 2.50), one nutritionist (full-time equivalent 0.50), two biomedical engineers, four health attendants and nine dialysis machines.

**Fig. 1:**
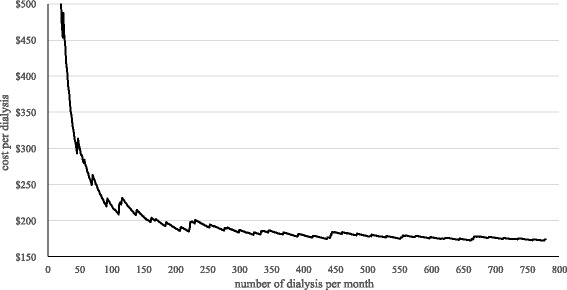
Average costs per dialysis

As discussed before the unit costs depend on the number of dialyses performed. Figure 1 shows the respective results.

Figure 1 clearly indicates that the average costs per dialysis strongly decrease with an increasing number of dialysis per month. The notable leaps show the influence of the step-fixed cost categories which cause higher costs if the maximum capacity of one step-fixed category is exceeded. Related to the fact that dialysis treatments are performed three times a week and 52 weeks per year, the average number of dialysis per patients and month is 13. It has to be considered that in case of a lower number of patients, the analysis also calculates with a lower need of personnel based on the mentioned capacities and working times on the dialysis unit. For example this would mean that the average cost per dialysis for a unit with 15 patients would be 192 US$, with 30 patients 178 US$, with 45 patient 177 US$ and with 60 patients 174 US$. Contrary to the cost range of these four examples a dialysis unit with only 3 patients would cause average costs of 331 US$ per dialysis. The reason for that is the increasing amount of fixed and step-fixed costs per costing unit in case of a lower number of dialysis per month.

**Fig. 2:**
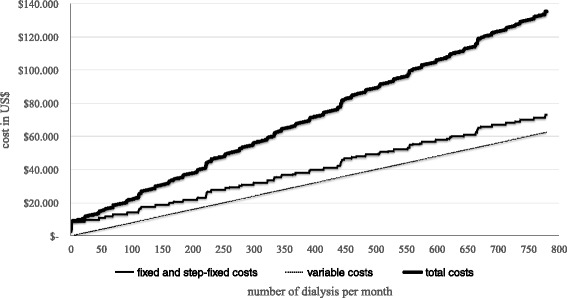
Monthly cost of the dialysis department

Figure 2 shows the total monthly unit costs as a sum of the total fixed costs (fixed and step-fixed) and the variable costs depending on the number of dialysis per month. Based on the analysis with nine machines at a number of 34 patients with an average of 442 dialysis per month the total monthly costs are 77,750 US$.

### Break-even analysis

**Fig. 3:**
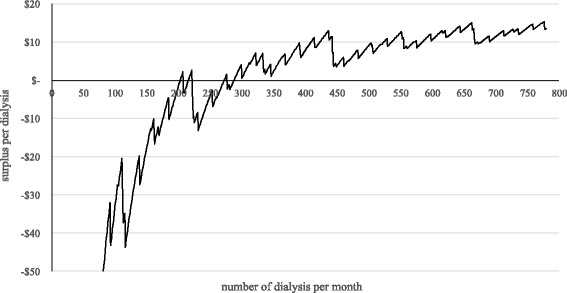
Surplus per dialysis

For patients with a public insurance the National Health Insurance Fund of Tanzania pays 187 US$ for a single HD. Relating to the results of Fig. 1 it is possible to calculate the average profit or loss per dialysis depending on the number of sessions per month. The results are shown in Fig. 3.

Figure 3 shows that a cost recovery is not given for every possible number of dialysis per month. Because of the influence of the step fixed costs, cost coverage is given in the areas between 203 to 207, 216 to 221 and 272 to 276 and above 288. To focus on these areas in case of planning the number of dialysis for the long run is not be advisable for a dialysis unit because of uncertainties in the future. The realistic point of break-even is at 288 dialyses. Beyond this number the department is able to cover the costs and to generate a surplus. Referring to the 13 dialyses per patient and month the number of 288 dialyses would be equivalent to 22.15 patients. Therefore, the minimum number of patients of an outpatient dialysis unit with only NHIF-insured patients should be at least 23. In case of the MNH the average surplus per dialysis is at 12 US$.

### Sensitivity analyses

The results presented above are subject to uncertainty of parameters. Therefore, we calculated the average cost per hemodialysis subject to different inputs (e.g. nutritionist, engineer, health attendants) and costs (e.g. rent, salaries, materials) of resources as well as different capacities of dialysis machines and personnel. Table 7 presents the results. The column “scenario” shows the parameters that are changed and the column “value of parameters” the new value. All other parameters are the standards given above (ceteris paribus). Unit costs are calculated for a utilization of 34 patients with 442 dialyses per month.

**Table 7:** Sensitivity Analysis

Scenario	Value of parameter	Average cost per dialysis [US$]
Basic	see Tables 2, 4, 5, 6	176
nutritionist	0 [number]	174
biomedical engineer	0 [number]	170
health attendant	0 [number]	173
nurse capacity	40 dialysis per month	155
rent	0 [US$ per month]	170
cost of dialyzer high flux	15 [S$ per dialysis]	166
cost of sterilization	5 [US$ per dialysis]	168
dialysis machines (depreciation and repair)	0 [US$ per month]	172
all personnel costs per category	−10 %	167
	−20 %	159
	−30 %	150
	−40 %	142
	−50 %	133
machine capacities/ personnel capacities	+25 % / +20 %	166
	+25 % / +40 %	156
	+50 % / +60 %	149
	+50 % / +80 %	146
	+50 % / +100 %	133
rent	0 Euro	131
nurse capacity	40 dialyses per month	
cost of dialyzer high flux	per dialysis 15 US$	
cost of sterilization	5 US$ per dialysis	

Source: own

It is obvious that lower unit cost per dialysis is possible if some cost categories are not included (e.g. rent), prices and salaries are lower (e.g. dialyzer high flux) and capacities are increased. A combined realistic scenario is presented in the last row. If rent is not included (because Muhimbili facilities are financed by the Government of Tanzania), if a nurse can handle up to 40 dialyses per month, if the dialyzer high flux costs on 15 US$ per dialysis and if the cost of sterilization is reduced to 5 US$ per dialysis the unit cost can decline to 131 US$.

## Discussion

CKD affects mainly young adults in their economically productive years aged 15–49. These findings are in consistence with other studies that reported an age of 20–50 years in Sub-Saharan Africa [25]. Contrary to this finding, in the developed world, CKD is present in the middle-aged and elderly patients and it’s predominantly due to diabetes nephropathy and hypertension [2].

The analysis has shown the average costs of a single dialysis and the total cost per month depending on the number of treatments. As Fig. 3 indicates, the hospital should perform at least 288 dialyses (equivalent to 22.15 patients) in order to break-even. However, if a dialysis patient needs erythropoietin – which is possibly not covered completely by revenues for the treatment of renal anemia – the dialysis surplus could be reduced by a kind of internal subsidization. This means that the surplus of dialysis treatments could be used for the compensation of possible losses in case of renal anemia treatments. For example, in case of the mentioned 34 patients at MNH 90 % needed erythropoietin with actual costs of 61.80 US$ per dialysis. This results in an average of 56 US$ per dialysis.

The results show that cost recovery in case of intermittent outpatient dialysis treatments is possible depending on the number of patients. According to latest information the number of patients under hemodialysis treatment has increased to 60 so that the average cost has declined to 174 US$. This is a tremendous increase and the respective figures indicate that the department is likely to generate a profit. However, it is not likely that the number of patients will increase even more as the department has come to its capacity limits. Firstly, nephrologists and specialized nurses are a rare category of staff in Tanzania. The cost curve assumes that more staff will be available but this is not guaranteed. Secondly, space in Muhimbili is scarce and a stronger increase of capacity would require more space which is most likely not available. Thirdly, there is doubt that the financing institutions are able and willing to sponsor many more patients. Thus, a strong increase in number is unlikely to happen.

At the same time, the results are based on the MNH at Dar es Salaam, a hospital of maximum treatment in the economic center and biggest agglomeration of Tanzania. It can be assumed that the results are partly transferable to dialysis units of larger hospitals in urban areas in this East African country. Transferability to providers in rural areas has to be doubted because of influencing factors like infrastructural conditions and a low population density. This means that dialysis centers in rural areas probably would have to calculate with higher average costs per treatment because of higher materials costs and with lower possible numbers of patients because of a lower population in the maximum catchment area. Therefore, an area-covering supply with renal replacement services in rural areas of developing countries appears to be very difficult to implement. Even though the majority of patients requiring dialysis are likely to live in urban places, still there are patients suffering from chronic kidney diseases in rural places – and it is very unlikely that they will find dialysis services there.

**Fig. 4:**
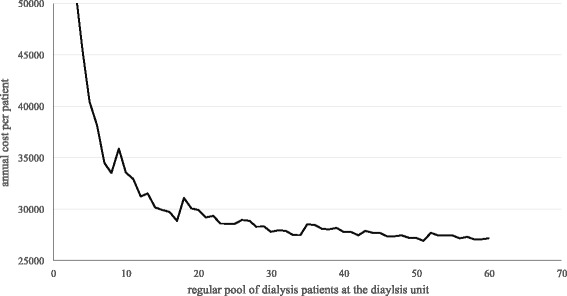
Annual cost per patient

In case of the dialysis unit at the MNH with 34 patients the average costs per dialysis is 176 US$. Assuming that a dialysis patient is treated three times a week, 52 week a year, the yearly number of dialysis is 156. Referring to this, the annual cost per patient is 27,440 US$ (see Fig. 4).

## Conclusion

In summary we can state that this cost analysis can provide the foundation of evidence based policy-making in the health care sector, efficient allocation of funds towards this specific service and proper calculation of fees. At the same time the cost of dialysis per patient per year is tremendous for a least developed country like Tanzania where resources are limited and materials have to be imported at high cost. Other diseases prevailing have a much higher burden of disease while being preventable and curable at much lower cost. Thus, ESRD and dialysis are a good example for the terrible dilemma which public health planners and policy-makers face in least developed countries: they have to allocate scarce resource to specific interventions. Neglecting dialysis patients will cause their death – but allocating high resource to them will mean that these resources will be missing for the fight against other diseases. The question “who shall live?” [26] is the terrible reality of policy-makers in Tanzania.

**Fig. 5:**
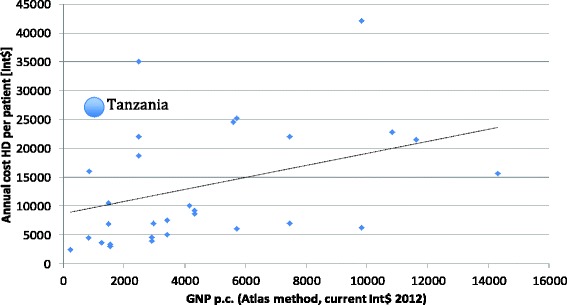
Annual cost of HD per patient in different countries. Source: own and [25]

However, before an allocation decision can be made every effort has to be invested to reduce the high actual cost by improving the technical efficiency of the dialysis departments. In order to assess the relative technical efficiency it is worthwhile to compare our results with cost of other low income countries. (Mushi L, Marschall P, Flessa S, The cost of dialyis in low- and middle-income come countries. Submitted.) calculated the annual cost per patient for hemodialysis and compared them with the gross national product. As Fig. 5 shows, the costs calculated in our study are higher than the expected value, in particular the cost of personnel were quite high (48 % of total cost). Contrary, Hooi et al., [27] found for Malaysia that the personnel cost consumes 19 % of total cost while consumables and drugs consume 26 % of all costs. In Sri Lanka, Ranasinghe et al. [20] found that drug and consumables costs accounted for 70–85 % of the total cost, followed by the wage of nursing staff 8 %–20 %.

In particular striking is the fact that the cost of dialyzer high flux in Tanzania is higher than in the developed world. It is beyond this study to address the issue of tariffs and taxes on medical equipment and materials, but before dialysis services are excluded from the basic health care package the issue of extraordinary materials cost has to be discussed within the country.

At the same time, the cost allocated to the dialysis unit for sterilization of medical equipment seems to be high. This figure was given by the hospital administration but it has to be questioned whether sterilization services could be obtained at a lower rate from outside the hospital. Furthermore, the highest fixed cost is the rent of the building. As this analysis calculates actual full costs it is appropriate to include it in the computation. However, it is unfair to compare the full cost of dialysis with the marginal cost of other services – and many cost analyses do not include rent or maintenance.

Finally, we have to question whether the staffing pattern was appropriate during our study period. Based on international standards this department was strongly over-staffed. It is beyond the scope of this paper to assess why such an input of nurses was needed per dialysis in Tanzania, but technical efficiency could be improved by reducing the nursing time per dialysis. In fact, the number of patients has increased in the meantime while staffing remained almost unchanged.

If we assume the assumptions of the combined scenario of Table 7 (no rent, nurse capacity 40 dialyses per month, cost of the dialyzer high flux per dialysis 15 US$, cost of sterilization 5 US$ per dialysis) the average cost per dialysis decline to 131 US$ and the total cost per patient per year to 20,500 US$. Thus, increased technical efficiency could buy one year of life with some 20,500 US$ - an amount that is still far beyond the traditional threshold of gross national product per capita.

One might argue that hemodialysis is a cost-intensive form of dialysis and peritoneal dialysis (PD) would be more adequate for a country like Tanzania. Currently, PD is not available in this East African country, but one might advise the Ministry of Health to foster the introduction of this technology. However, the literature on the cost of PD in less and least developed countries is even more scarce than for HD and it does not strongly support the assumption that PD is always less expensive than HD. It holds true for developed countries which manufacture PD solution themselves. In some countries the costs seem to be equal, e.g. in China [28] and Namibia [7]. In other of countries the cost of PD are reported higher than the cost of HD, for instance for Sri Lanka HD [28], Bangladesh [28] and Sudan [7]. Consequently, Tanzania which has to import PD solutions completely would have to make a thorough analysis before decisions could be done.

Consequently, the findings of this study indicate that hemodialysis services in Tanzania are a very resource intensive technology in comparison to the health care budget of the nation. It seems more rational to allocate health care budgets towards diseases that can be prevented, cured or treated at lower costs. But the people of Tanzania should decide themselves in a transparent political process on their health care package. Political decisions are influenced by many factors, not only economic evidence [29]. However, the knowledge of provider cost is one cornerstone of transparency in this endeavour but more research on household cost, quality of life of dialysis patients, quality of services, effectiveness, efficiency and alternatives to dialysis is needed to base allocation decisions on evidence.
